# Powder Handling and Frozen Texture of a Reconstitutable Strawberry–Oat Sorbet Premix After Sucrose Replacement

**DOI:** 10.1111/jtxs.70124

**Published:** 2026-10-14

**Authors:** Caroline dos Santos Lima, Suzana Caetano da Silva Lannes

**Affiliations:** ^1^ Department of Biochemical‐Pharmaceutical Technology, School of Pharmaceutical Sciences University of São Paulo (USP) São Paulo Brazil

**Keywords:** back extrusion, frozen dessert, inulin, maltodextrin, melting behavior, powder flowability

## Abstract

A dry sorbet premix must remain manageable as a powder and still develop an appropriate structure after reconstitution and freezing. This study compared a sucrose‐containing control with a strawberry–oat premix in which sucrose was replaced by maltodextrin, inulin, and a commercial high‐intensity sweetener blend. Three independently prepared batches were evaluated in the dry state and after reconstitution and dynamic freezing. The sucrose‐replaced powder had a higher angle of repose (44° vs. 39°) and a lower compaction force (2.65 vs. 7.65 N); both powders wetted in approximately 4 s. It also contained modestly more total dietary fiber (4.76% vs. 4.27%), whereas the reported protein and carbohydrate‐by‐difference estimates did not differ statistically. The prepared sucrose‐replaced sorbet required a higher back‐extrusion force (6.50 vs. 4.25 N), retained a similar overrun (approximately 50%), and showed less drainage in the available melting profile. The composition and texture results were considered together, but no correlation coefficient was calculated because only two formulations were studied and replicate‐level rheology and melting series were unavailable. Replacing sucrose therefore created a powder–texture trade‐off; the findings do not establish nutritional superiority or isolate individual ingredient effects.

## Graphical abstract

Development and production workflow of the reconstitutable strawberry–oat sorbet premix, from ingredient preparation and formulation design through dry blending, reconstitution, dynamic freezing, and final product evaluation. The schematic summarizes the experimental process and the principal groups of analyses performed.

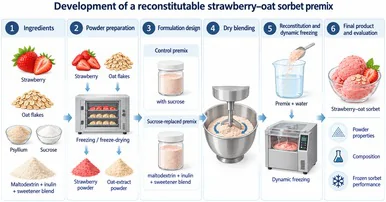

## 1 Introduction

Sorbet is a fruit‐based, nondairy frozen dessert in which ice crystals and air cells are dispersed within a freeze‐concentrated aqueous phase containing fruit solids, sugars or sweeteners, and stabilizing ingredients. Unlike dairy ice cream, its structure is not supported by a fat–protein network; consequently, the amount and type of soluble solids and hydrocolloids strongly influence hardness, melting, and perceived body (Goff and Hartel 2013; Freire et al. 2025).

Reducing added sucrose is relevant to health‐oriented product development because high dietary sugar exposure has been associated at the population level with adverse outcomes. This context motivates reformulation research but does not establish that the present prototype is healthier: total sugars, energy value, glycemic response, intake, and clinical outcomes were not measured (Huang et al. 2023). Technologically, sucrose provides more than sweetness. It contributes bulk and soluble solids, depresses the freezing point, changes the amount of unfrozen water and the viscosity of the serum phase, and thereby affects ice formation, deformation, and drainage during melting. High‐intensity sweeteners alone cannot replace these functions, so bulking and water‐structuring ingredients are normally required (Liburdi et al. 2025; Long et al. 2024; Wang et al. 2025).

Strawberry was selected as a technologically informative fruit matrix rather than only as a flavor. Freeze‐dried strawberry contributes acids, suspended solids, pectin, and dietary fiber that can alter hydration and melting, while freeze‐drying conditions materially influence the resulting fruit powder (Biernacka et al. 2022; Kocaman et al. 2025). Strawberry powder has been reported to increase mix viscosity while reducing hardness and meltdown in frozen dairy desserts, indicating that strawberry solids can materially affect frozen‐dessert structure (Bilbao‐Sainz et al. 2019). The acidic red‐fruit matrix also provides a relevant test of whether a reconstitutable powder can retain recognizable product identity after drying and freezing.

A filtered oat extract was used to provide a plant‐derived aqueous phase and oat‐derived soluble and suspended solids without the coarse insoluble load of whole oat flour. Oat‐based frozen desserts are sensitive to the concentration and functionality of their continuous‐phase solids, and oat ingredients have been associated with changes in rheology, overrun, melting resistance, and sensory texture (Buniowska‐Olejnik et al. 2023; Henden et al. 2024). In the present work, the filtered extract was freeze‐dried separately to create a storable ingredient that could be dry‐blended and reconstituted with the other components. No claim is made about β‐glucan concentration because it was not measured.

Previous research has usually approached either ready‐to‐freeze sugar‐reduced mixes or the rehydration of food powders. Far fewer studies have followed the same fruit‐based formulation across dry handling, reconstitution, dynamic freezing, instrumental deformation, and melting. This cross‐state comparison is the specific contribution of the present work: it tests whether improving frozen‐product resistance after sucrose replacement introduces a handling penalty at the powder stage (Jiang et al. 2025; Suhag et al. 2024; Wang et al. 2025).

Accordingly, this study compared a sucrose‐containing control with a sucrose‐replaced strawberry–oat sorbet premix. The objectives were to quantify selected powder properties, reconstitution performance, back‐extrusion force, overrun, and melting behavior, and to identify trade‐offs between the dry and frozen states. It was hypothesized that the maltodextrin–inulin replacement would preserve bulk and increase frozen‐product resistance while changing particle‐level flow and compaction. Because all replacement ingredients changed simultaneously, the study was designed as a formulation‐level proof of concept and not as an attribution of effects to individual ingredients.

## 2 Materials and Methods

### 2.1 Materials and Ingredient Functions

Food‐grade frozen strawberry (*Fragaria × ananassa* Duch.; Dmarchi, Jundiaí, SP, Brazil), medium oat flakes (Granolevis, Atibaia, SP, Brazil), psyllium ( *Plantago ovata* ; Vitacel Psyllium P95, JRS Latinoamérica, Diadema, SP, Brazil), sucrose (União, São Paulo, SP, Brazil), corn maltodextrin (MOR‐REX 1920, Ingredion, São Paulo, SP, Brazil; nominal dextrose equivalent 20, supplier specification 17.0–19.9), inulin (Orafti HSI, Beneo, São Paulo, SP, Brazil), and a commercial culinary sweetener (Linea Sucralose Forno & Fogão, Goiânia, GO, Brazil) were used. Photographs of the product label confirmed the ingredient declaration “maltodextrin; sweeteners: sucralose and acesulfame potassium.” The ingredient is therefore identified as a commercial sweetener blend rather than as pure sucralose.

Maltodextrin supplied bulk and solids but was not treated as a dietary fiber. Inulin supplied bulk and fermentable fiber, while the commercial blend adjusted sweetness. Psyllium was held constant in both formulations as a hydrated structuring ingredient. These ingredients were selected to address separate functions lost when sucrose was removed; they were not assumed to reproduce sucrose molecularly (Goff and Hartel 2013; Guo et al. 2009; Wang et al. 2025).

### 2.2 Formulation Design

The replacement proportions were selected after bench‐scale pilot trials that screened mixing feasibility, rapid visible wetting, sweetness acceptability, and the ability to produce a self‐supporting frozen dessert. The trials were not a formal response‐surface optimization, and the two formulations were not demonstrated to be equisweet by sensory testing. Original formulation records were expressed as mass parts and summed to 102.0 and 100.2; Table 1 reports the same ratios normalized to 100.0% to remove rounding ambiguity.

**TABLE 1:** Normalized composition of the dry premixes.

Ingredient	Control (% w/w)	Sucrose‐replaced (% w/w)
Freeze‐dried strawberry	25.2	25.2
Freeze‐dried filtered oat extract	14.0	13.7
Psyllium	2.0	2.0
Sucrose	58.8	—
Maltodextrin	—	48.2
Inulin	—	9.2
Commercial sweetener blend	—	1.7
Total	100.0	100.0

*Note:* Percentages were normalized from the original mass‐part ratios (control: 25.7, 14.3, 2.0, and 60.0; sucrose‐replaced: 25.3, 13.7, 2.0, 48.3, 9.2, and 1.7, respectively). Photographs of the commercial product label confirmed that the sweetener blend contained maltodextrin, sucralose, and acesulfame potassium.

### 2.3 Preparation and Freeze‐Drying of the Oat Extract and Strawberry

Oat flakes were hydrated in filtered water (1:4, w/w) for 16 h under refrigeration, processed for 5 min in a domestic processor (PH900, Philco, Brazil), and passed through a fine‐mesh sieve. Filtration was chosen instead of direct use of oat flour to reduce coarse insoluble particles that could impair dispersion and produce a gritty frozen matrix. The liquid extract was distributed in Petri dishes and frozen at −65°C for 24 h.

Strawberries were cut into small cubes, distributed in Petri dishes, and frozen at −65°C for 24 h. Oat extract and strawberry were freeze‐dried separately in an FTS System TDS‐00209A freeze‐dryer (FTS Systems, USA). The confirmed laboratory record reports an initial 10‐min stage at −40°C and 750 Torr, followed by 960 min at −25°C and 100 mTorr. Because 750 Torr is close to atmospheric pressure, the first stage is described operationally as an initial temperature‐conditioning step rather than as primary drying under vacuum. Product‐temperature histories and a documented drying‐endpoint criterion were not retained; therefore, the cycle is reported as executed and is not presented as optimized or validated. Although developed primarily for pharmaceutical lyophilization, process‐analytical studies have demonstrated the complementary value of product‐temperature measurements, pressure‐based monitoring, and endpoint‐detection methods for evaluating and controlling freeze‐drying processes (Fissore et al. 2018; Pisano 2022). Freeze‐dried ingredients were placed in capped glass containers, protected with plastic film and aluminum foil, and stored at 7.5°C until use. Separate drying was used to create two dry‐blendable ingredients for subsequent reconstitution (Biernacka et al. 2022; Nowak and Jakubczyk 2020; Prosapio and Lopez‐Quiroga 2020; Waghmare et al. 2022).

### 2.4 Size Reduction and Powder Blending

Sucrose and freeze‐dried strawberry were ground for 5 min at speed 1 in a domestic processor (PH900, Philco, Brazil); freeze‐dried oat extract was briefly homogenized with the pulse function. Psyllium, maltodextrin, inulin, and the commercial sweetener blend were used as supplied. Ingredients were blended in a drum mixer (Indústria Paulista de Motores, three‐phase motor 26,021, type T56, Brazil) for 10 min.

Mixing time was selected in an operational pilot adapted from Fellows (2009). A 100‐g complete premix, including freeze‐dried strawberry, was blended for 5 min. Four 10‐g subsamples were then shaken for 5 min at speed 8 in a Granutest classifier (Produtest, Brazil) fitted with an ABNT 25/Tyler 24 sieve. The retained masses were 3.4, 3.7, 3.5, and 3.6 g. The subsample standard deviation (s_m_), mixing index (M_1_), initial standard deviation (s_0_), and first‐order mixing constant (K) were defined as follows:

(1) $$s_{m}=\sqrt{\frac{\sum_{i=1}^{n}{\left(c_{i}-\bar{c}\right)}^{2}}{n-1}}$$

(2) $$M_{1}=\frac{s_{m}-s_{\infty }}{s_{0}-s_{\infty }}$$

(3) $$s_{0}=\sqrt{V_{1}\left(1-V_{1}\right)}$$

(4) $$\ln\left(M_{1}\right)=-Kt_{m}$$

where *c*_i_ is the mass retained for subsample *i* (g), *c̄* is the mean retained mass (g), n is the number of subsamples, *s*∞ is the standard deviation assumed for a perfectly mixed system, *V*_1_ is the mass fraction of the tracer component, M_1_ is dimensionless, *K* is the first‐order mixing constant (s^−1^), and *t*_*m*_ is the mixing time (s). The archived worksheet did not retain the numerical values assigned to *s*∞ and *V*_1_ or the intermediate *M*_1_ and *K* results; therefore, the previously stated 9.93‐min value cannot be independently recalculated and is not repeated as an exact result. The retained operational conclusion was a minimum of approximately 10 min, and both production blends were mixed for 10 min. This pilot served only to select the executed operating condition and was not treated as a validated blend‐uniformity result (Barbosa‐Cánovas et al. 2005; Fellows 2009).

### 2.5 Powder Particle Size, Wettability, Angle of Repose, and Compaction

Particle‐size distribution was determined with a Granutest sieve analyzer (Produtest, Brazil). A 10‐g sample was shaken for 5 min at speed 8. The control was evaluated with ABNT/Tyler 30/28, 50/48, 60/60, 80/80, 100/100, and 140/150 sieves; the sucrose‐replaced powder used ABNT/Tyler 20/20, 30/28, 40/35, 50/48, 80/80, and 170/170 sieves. Different stacks were selected in preliminary trials to prevent excessive retention in the upper sieve or pan. The weighted mean particle diameter (Dm, mm) was calculated as:

(5) $$D_{m}=\frac{\sum_{i}R_{i}A_{i}}{100}$$

where *R*_*i*_ is the mass retained in fraction *i* (%), and Ai is the mean opening of the two sieves that bound fraction *i* (mm) (Barbosa‐Cánovas et al. 2005).

Wettability was measured with a modification of Lannes and Medeiros (2003). Powder (2.0 g) was distributed uniformly from a height of 3 cm onto 50 mL of filtered water at 25°C. The time until the powder surface was completely covered by liquid was recorded in seconds. This visual endpoint describes initial wetting, not complete dissolution or hydration (Fang et al. 2008).

For the angle of repose, 20 g of powder flowed through a funnel with a 10‐mm outlet onto a Petri dish positioned 2 cm below the funnel. Cone height (h, cm) and base diameter (D, cm) were measured, and the angle (θ, degrees) was calculated as:

(6) $$\theta =\arctan\left(\frac{2h}{D}\right)$$

Powder compaction was determined using a TA‐XT2 texture analyzer (Stable Micro Systems, UK) using a procedure adapted from Eduardo and Lannes (2007). Powder (40 g) was placed in a cylindrical glass vessel (50‐mm internal diameter; 70‐mm height) and compressed with a 45‐mm plate at 2 mm·s^−1^ for 5 s over a distance of 2 mm. The maximum force was reported in newtons.

### 2.6 Selected Composition and Water Activity

Moisture was determined gravimetrically at 105°C to constant mass (AOAC 931.04), ash by incineration in a muffle furnace at 550°C to constant mass, and total dietary fiber by an enzymatic–gravimetric procedure using heat‐stable α‐amylase, protease, and amyloglucosidase (AOAC 985.29). Water activity was measured at 25°C with a LabMaster.aw meter (Novasina, Switzerland) only for the separately freeze‐dried ingredients. The values 0.32 and 0.05 corresponded to strawberry and oat‐extract powders, respectively, and were not treated as control‐versus‐replacement formulation results.

The available analytical summary also reported protein determined by a Micro‐Kjeldahl procedure and carbohydrates by difference. The nitrogen‐to‐protein conversion factor could not be recovered from the laboratory record; protein is therefore identified as a reported estimate rather than a fully reproducible nutritional determination. The reported carbohydrate remainder was calculated as:

(7) $${\hat{C}}_{\text{diff}}=100-\left(M+A+P+L\right)$$

where ${\hat{C}}_{\text{diff}}$ is carbohydrate by difference (%), *M* is moisture (%), *A* is ash (%), *P* is reported protein (%), and *L* is lipid (%). Lipid was not measured and was entered as zero in the archived calculation. Consequently, the reported value is an incomplete remainder that includes dietary fiber; it is not a measurement of available carbohydrate, total sugars, or glycemic carbohydrate. Protein and carbohydrate estimates were retained only to contextualize formulation composition and were not used to support nutritional claims (AOAC International 2019).

### 2.7 Reconstitution and Dynamic Freezing

Each premix (215 g) was dispersed in 500 mL of filtered water and mixed for 5 min with a hand mixer (Ultra Mixer 1800 W, Britânia, Brazil). The reconstituted mix was processed for 40 min in a pre‐frozen batch ice‐cream maker (ICE‐20, Cuisinart, USA) to promote partial freezing and air incorporation. Back‐extrusion and overrun were measured immediately after churning. Samples used for the melting test were subsequently held at −20°C; samples for other stored measurements were hardened at −18°C for 24 h.

### 2.8 Exploratory Oscillatory Rheology

Reconstituted mixes from independently prepared batches were evaluated with a MARS Haake rheometer (Thermo Electron Corporation, Germany) equipped with a polished C35/1 Ti cone–plate sensor and controlled with RheoWin3 software. Temperature was maintained at 4°C. Stress sweeps were conducted from 0.01 to 100 Pa, followed by archived frequency sweeps reported over 0.01–10 Hz. Strawberry particles and seeds produced irregular stress‐sweep responses, and a stable linear viscoelastic region could not be verified. The original rheometer files are no longer available; therefore, the retained mechanical‐spectrum graph was used only as an exploratory visual record. No inferential comparison, fitted rheological parameter, definitive material classification, or causal interpretation was based on these curves.

### 2.9 Back Extrusion, Overrun, and Melting

Instrumental resistance of the prepared sorbet was measured immediately after churning at −5°C with a CTX texture analyzer (AMETEK Brookfield, USA) and an AB/E back‐extrusion probe. Cups were filled to two‐thirds of their volume. Pre‐test, test, and post‐test speeds were 1 mm·s^−1^, and the probe traveled 25 mm after contacting the sample. The maximum force (Fmax, N) was extracted using Texture Pro V 1.0 Build 15 software.

Overrun was calculated from the masses of the same fixed volume of unfrozen mix (mmix, g) and prepared sorbet (msorbet, g):

(8) $$\text{Overrun}\;\left(\%\right)=\frac{m_{\mathrm{mix}}-m_{\text{sorbet}}}{m_{\text{sorbet}}}\times 100$$

For the melting test, an adapted drip‐through procedure based on (Bolliger et al. 2000) was used. Samples (30 g), conditioned at −20°C, were placed on a metal grid for 45 min at a controlled room temperature of 24°C, and cumulative drained mass (Mt, g) was recorded throughout the test.

(9) $$M_{t}=W_{t}-W_{0}$$

(10) $$\text{Cumulative drainage}\;\left(\%\right)=\frac{M_{t}}{M_{0}}\times 100$$

where *W*_*t*_ is the mass of the collection vessel plus drained sample at time *t* (g), W0 is the empty‐vessel mass (g), and M0 is the initial sorbet mass (g). Because the replicate‐level numerical time series was not included in the supplied dataset, the archived mean profiles were interpreted descriptively and were not subjected to inferential testing.

### 2.10 Experimental Replication and Statistical Analysis

Three independently prepared batches of each formulation were used for every analysis (*n* = 3 independent experimental units per formulation). Measurements from different batches, rather than repeated instrument readings from the same preparation, constituted the replicates. Results are reported as the available mean ± standard deviation. Because two formulations were compared, the Welch two‐sample *t* statistic and Satterthwaite degrees of freedom were calculated directly from the reported n, mean, and standard deviation for each formulation:

(11) $$\mathrm{SE}=\sqrt{\frac{s_{1}^{2}}{n_{1}}+\frac{s_{2}^{2}}{n_{2}}}$$

(12) $$t=\frac{{\bar{x}}_{1}-{\bar{x}}_{2}}{\mathrm{SE}}$$

(13) $$\nu =\frac{{\left(\frac{s_{1}^{2}}{n_{1}}+\frac{s_{2}^{2}}{n_{2}}\right)}^{2}}{\frac{{\left(s_{1}^{2}/n_{1}\right)}^{2}}{n_{1}-1}+\frac{{\left(s_{2}^{2}/n_{2}\right)}^{2}}{n_{2}-1}}$$

where *x̄*, *s*, and *n* are the reported mean, standard deviation, and number of independent batches for each formulation; SE is the standard error of the difference and *ν* is the Satterthwaite approximation to the degrees of freedom. Two‐sided *p* values were obtained from the *t* distribution at *α* = 0.05. With only three independent batches per formulation, formal normality testing would have had little diagnostic value and could not be revisited from the retained summary statistics. No equal‐variance assumption was imposed; Welch's test was therefore used for endpoints with nonzero reported dispersion. No individual observations were reconstructed. Inferential testing was not performed when both reported standard deviations were zero, when dispersion was unavailable, or when the only retained evidence was an archived graph; these endpoints were marked not calculated (NC) or discussed descriptively.

Composition, rheology, back‐extrusion, overrun, and melting were integrated by comparing the direction and magnitude of the formulation‐level patterns. Pearson or Spearman correlation coefficients were not calculated because the design contained only two formulations and replicate‐matched numerical rheology and melting series were unavailable. The integrated interpretation is therefore a triangulation of complementary endpoints, not a statistical correlation analysis.

Figure 1 summarizes the two formulations, the processing sequence, and the endpoints evaluated from the dry premix to the prepared sorbet.

**FIGURE 1:**
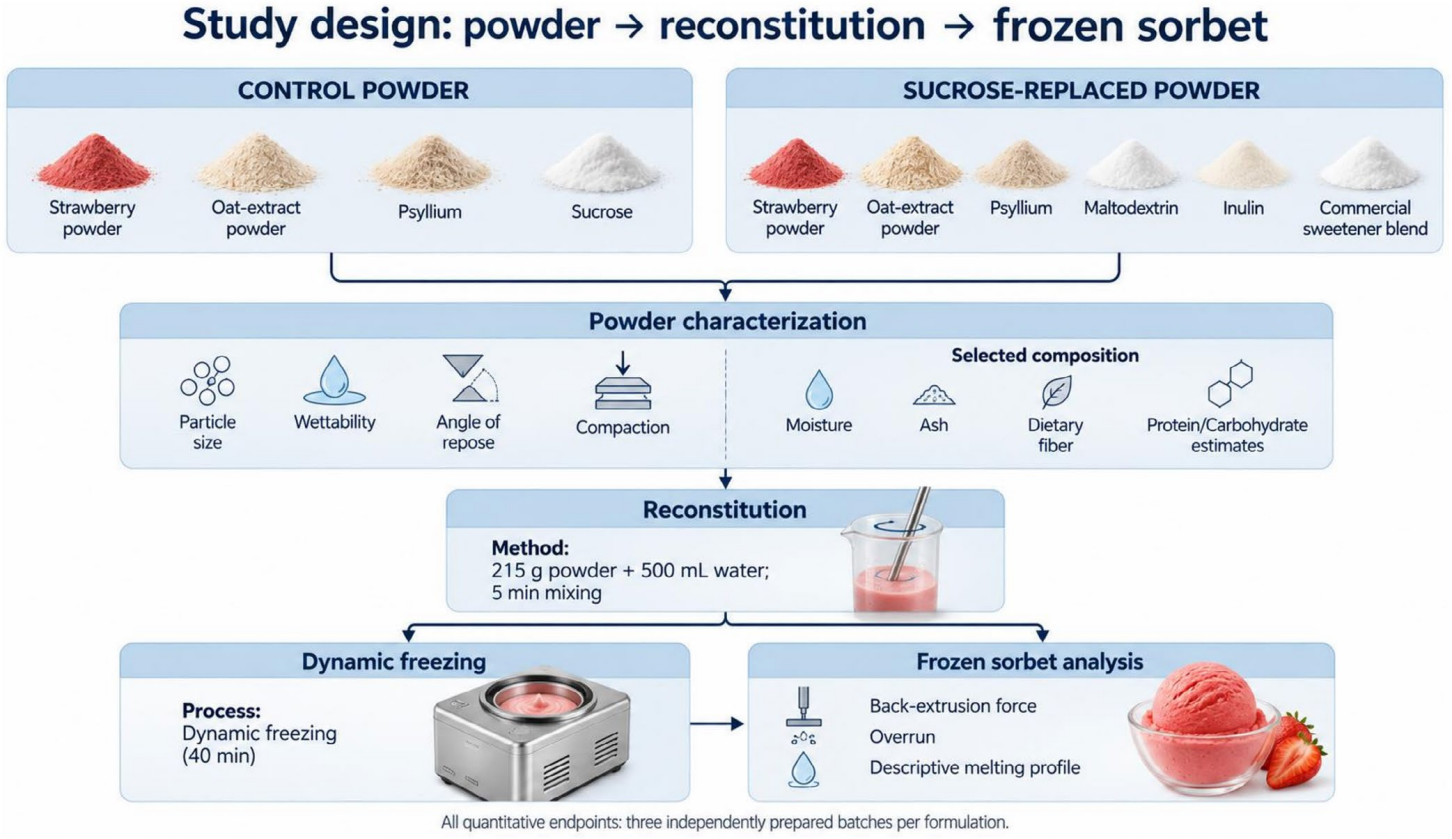
Study design from powder to prepared sorbet. The diagram gives the normalized formulation percentages and the endpoints evaluated at each stage. It is a communication schematic and does not depict measured microstructure or a causal mechanism. All quantitative endpoints were obtained from three independently prepared batches per formulation.

## 3 Results and Discussion

### 3.1 Powder Composition, Handling, and Reconstitution

The sucrose‐replaced powder had higher moisture (0.85% ± 0.02%) than the control (0.52% ± 0.04%; *p* = 0.001) and a modestly higher total dietary fiber content (4.76% ± 0.21% vs. 4.27% ± 0.08%; *p* = 0.042; Table 2). Because formulation‐level water activity was not measured, no inference is made about water binding or shelf stability. The ingredient‐level water‐activity values reported in Section 2.6 only confirm that the separately dried strawberry and oat extract differed in residual water availability.

**TABLE 2:** Selected composition and physical properties of the dry premixes.

Endpoint	Control, mean ± SD	Sucrose‐replaced, mean ± SD	*p*
Moisture (%)	0.52 ± 0.04	0.85 ± 0.02	0.001
Ash (%)	1.29 ± 0.00	1.39 ± 0.00	NC
Total dietary fiber (%)	4.27 ± 0.08	4.76 ± 0.21	0.042
Reported protein estimate (%)	0.03 ± 0.00	0.14 ± 0.10	0.197
Carbohydrate by difference (%)	98.16 ± 1.00	97.62 ± 1.00	0.545
Mean particle diameter (mm)	0.227 (SD NR)	0.211 (SD NR)	NC
Angle of repose (°)	39.00 ± 0.00	44.00 ± 0.02	< 0.001
Wettability (s)	3.99 ± 0.00	3.98 ± 0.00	NC
Compaction force (N)	7.65 ± 0.00	2.65 ± 0.00	NC

*Note:* Reported mean ± standard deviation (*n* = 3 independent batches). Welch's two‐sided *t*‐test was calculated directly from n, mean, and standard deviation; individual observations were not reconstructed. Protein is a retained estimate because the nitrogen‐to‐protein conversion factor was unavailable. Carbohydrate by difference is an incomplete remainder because lipid was not measured and was entered as zero; it includes dietary fiber and is not available carbohydrate or total sugars. NC, not calculated because both groups showed no measurable dispersion at the available precision or because exact dispersion was unavailable.

The low moisture values of the final premixes describe the products obtained; they do not, by themselves, validate the freeze‐drying cycle. Chamber pressure, product temperature, heat transfer, and dried‐layer resistance act together during drying. Process‐analytical literature emphasizes that freeze‐drying cycle evaluation should not rely on chamber pressure alone, but should consider complementary information from product‐temperature monitoring and appropriate endpoint‐detection methods (Fissore et al. 2018; Pisano 2022). Those records were not available here. The present interpretation is therefore limited to the measured final moisture and is consistent with recent food and strawberry studies showing that drying conditions can alter kinetics and powder quality (Biernacka et al. 2022; Kocaman et al. 2025; Waghmare et al. 2022). Moisture content and water activity are complementary rather than interchangeable because water desorption depends on the specific interactions between water and the solid matrix (Barbosa‐Cánovas and Juliano 2020).

Mean particle diameter was similar in magnitude for the two fine powders (0.227 mm for the control and 0.211 mm for the sucrose‐replaced formulation). The 7% numerical difference was interpreted descriptively because no dispersion was available at the reported precision. Particle size alone did not predict handling: the sucrose‐replaced powder had a higher angle of repose (44.00° ± 0.02° vs. 39.00° ± 0.00°; *p* < 0.001), indicating poorer gravity‐driven flow under the funnel test. Replacing crystalline sucrose with maltodextrin and inulin changed the formulation composition and may also have altered particle packing, while the slightly finer distribution may have increased interparticle contacts. These are formulation‐level associations; particle shape, density, and hygroscopicity were not measured (Juliano and Barbosa‐Cánovas 2010; Suhag et al. 2024).

Both powders wetted rapidly (approximately 4 s), showing that the replacement system did not delay the initial visual wetting endpoint. However, wetting should not be equated with complete dissolution or hydration in a matrix containing psyllium, strawberry solids, and oat‐derived material (Fang et al. 2008; Jiang et al. 2025).

Compaction force decreased from 7.65 to 2.65 N. This endpoint was interpreted descriptively because both standard deviations rounded to zero. The opposite directions of the angle of repose and compaction show that unconfined flow and confined compression are not interchangeable powder properties: a cohesive powder can flow poorly through a funnel yet rearrange readily under a plate (Eduardo and Lannes 2007; Juliano and Barbosa‐Cánovas 2010). For premix manufacturing, the practical implication is a trade‐off between rapid reconstitution and less favorable dosing flow.

### 3.2 Exploratory Rheological Observation

The archived oscillatory rheology profiles of the reconstituted mixes are presented in Figure 2.

**FIGURE 2:**
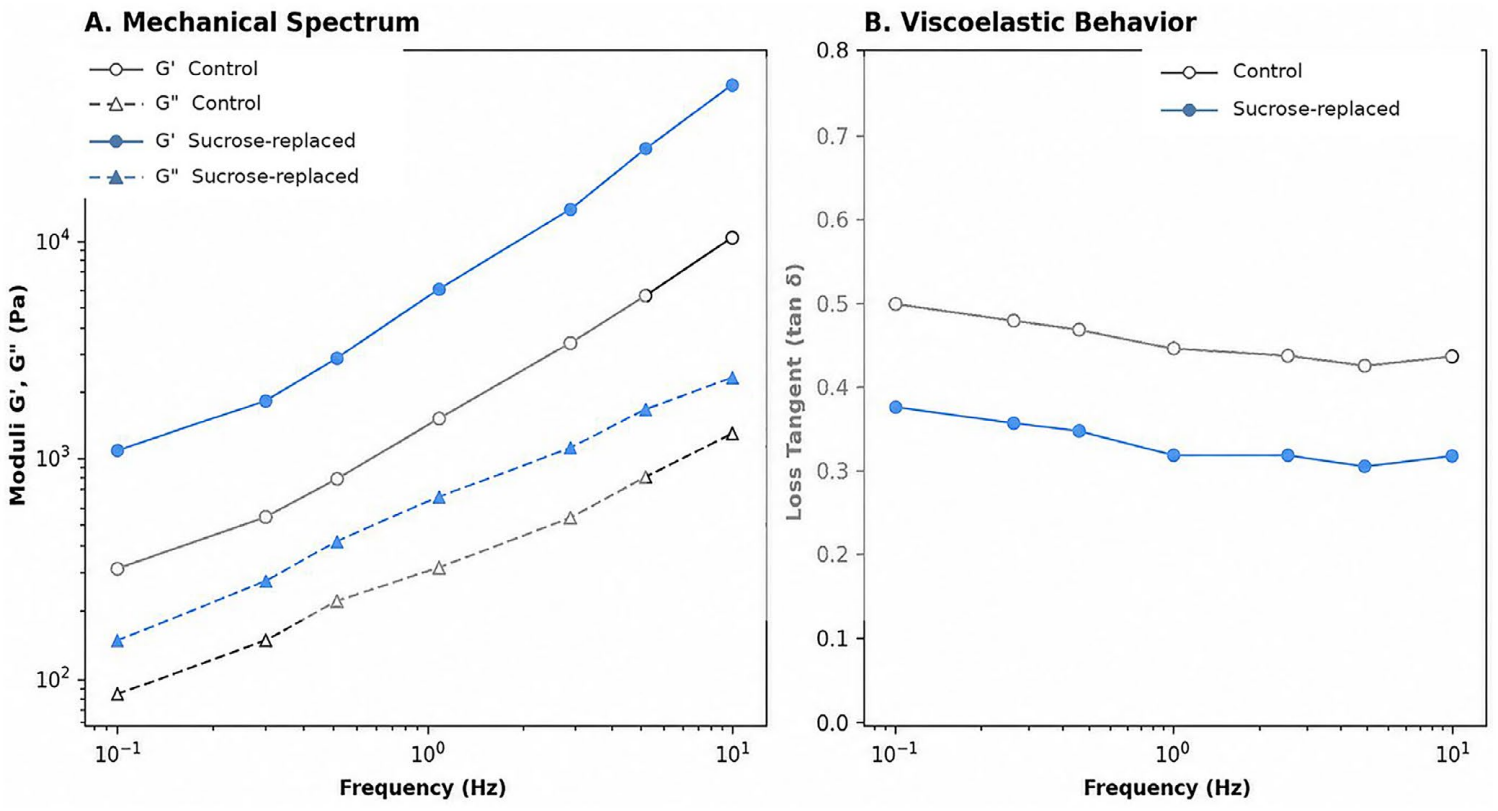
Archived exploratory oscillatory rheology of the reconstituted mixes. Panel A shows *G*′ and *G*″, and Panel B shows tan *δ*. The color palette and formulation label were standardized for consistency, but curve geometry and plotted values were not altered. The curves are descriptive because raw files, replicate‐level values, and a verifiable linear viscoelastic region were unavailable.

In the available graph, *G*′ was plotted above *G*″ for both formulations and tan δ remained below 1 across the displayed frequencies; the sucrose‐replaced formulation also showed higher plotted moduli and lower plotted tan δ than the control (Figure 2). These observations apply only to the archived curves. Because the original rheometer files and replicate‐level curve values could not be recovered, and because a stable linear viscoelastic region was not verified, uncertainty, fitted parameters, and inferential comparisons cannot be reconstructed. The curves therefore do not establish a weak‐gel classification or a causal explanation for back extrusion and melting. Confirmatory rheology would require the analysis to be repeated with particle‐appropriate geometry, a validated gap, a documented stress within the linear region, and preserved raw files.

### 3.3 Instrumental Frozen‐Sorbet Performance

Back‐extrusion force increased from 4.25 ± 0.02 N in the control to 6.50 ± 0.03 N in the sucrose‐replaced sorbet (*p* < 0.001; Table 3). This is the clearest texture result: under the standardized test conditions, the replacement formulation offered 53% greater resistance to probe displacement. Sucrose removal can increase the frozen‐water fraction when freezing‐point depression is not fully restored, while maltodextrin, inulin, psyllium, and oat/strawberry solids can change the unfrozen phase. Because neither freezing point nor ice‐crystal size was measured, the relative contributions of ice and continuous‐phase solids cannot be separated (Goff and Hartel 2013; Muse and Hartel 2004; Wang et al. 2025). Recent studies of sucrose‐reduced or fiber‐containing ice creams likewise show that reformulation can change hardness, rheology, melting, and sensory response together; however, their dairy matrices and designs are not directly equivalent to the present sorbet (Gözübenli and Yaşar 2026; Liburdi et al. 2025).

**TABLE 3:** Instrumental texture and overrun of the prepared sorbets.

Endpoint	Control, mean ± SD	Sucrose‐replaced, mean ± SD	*p*
Back‐extrusion force, Fmax (N)	4.25 ± 0.02	6.50 ± 0.03	< 0.001
Overrun (%)	49.99 ± 0.00	50.91 ± 0.00	NC

*Note:* Reported mean ± standard deviation (*n* = 3 independent batches). Welch's test was calculated from the summary statistics. NC, not calculated because both groups showed no measurable dispersion at the available precision. The melting profile was descriptive and is therefore discussed in the text rather than summarized with inferential statistics.

The direction of the firmness response differs from the decrease in hardness reported when strawberry powder alone was added to a dairy dessert (Bilbao‐Sainz et al. 2019). That contrast is expected because the present study simultaneously replaced 58.8% sucrose with several solids in a nondairy sorbet premix. It underscores that strawberry powder, oat solids, inulin, maltodextrin, psyllium, and sweetener composition must be interpreted as a combined formulation rather than as isolated factors.

Overrun remained close to 50% in both formulations. With standard deviations rounding to zero, the 0.92‐percentage‐point numerical difference was not treated inferentially. The result indicates that air incorporation was maintained under the small‐batch process; it does not establish air‐cell size or stability, which require microscopy (Freire et al. 2025).

Figure 3 presents the archived cumulative‐drainage profiles recorded at 24°C.

**FIGURE 3:**
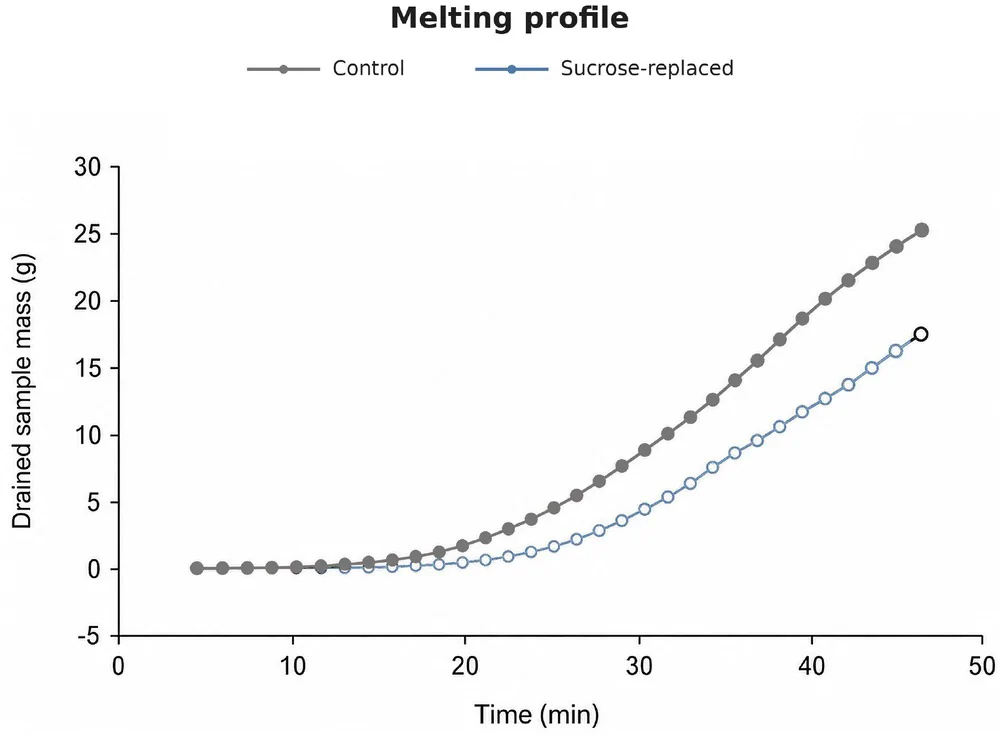
Archived melting profile of the prepared sorbets at 24°C. Drained mass was recorded throughout the 45‐min test using 30‐g samples. The color palette and formulation label were standardized for consistency, but curve geometry and plotted values were not altered. Because replicate‐level time‐series values were unavailable, the curves are shown without error bars or inferential comparison and were not used to calculate a melting‐rate parameter.

At 24°C, the archived melting profile showed later and lower drainage for the sucrose‐replaced sorbet than for the control (Figure 3). Because the replicate‐level numerical series was not available in the supplied dataset, the profile is evidence of direction only and no melting‐rate parameter or *p* value was calculated. Instrumental firmness and drainage therefore converge at the formulation level, but they do not demonstrate a causal mechanism. Recent work on nondairy frozen desserts and fiber‐containing ice creams likewise shows that melting depends on multiple structural variables and that a single rheological or textural metric cannot represent every phase of meltdown (Freire et al. 2025; Gözübenli and Yaşar 2026).

### 3.4 Novelty, Interpretation, and Study Boundaries

The contribution of this study does not lie in using maltodextrin, inulin, or strawberry individually. It lies in following the same reconstitutable formulation across two use states and revealing a trade‐off that would otherwise be easy to miss (Tables 2 and 3; Figure 3). The sucrose‐replaced powder flowed less readily under gravity, yet the prepared sorbet resisted instrumental deformation more strongly and showed lower drainage in the archived melting profile. A study confined to the liquid mix or final dessert would overlook the handling penalty, whereas a powder‐only study would miss the frozen‐texture response.

The design nevertheless supports only a formulation‐level comparison. Six component proportions differed between the two blends, so the effects of sucrose removal cannot be separated from those of maltodextrin, inulin, the commercial sweetener carrier, or the small differences in strawberry and oat‐extract proportions. The study also lacked freezing‐point measurements, direct ice‐crystal and air‐cell imaging, validated confirmatory rheology, sensory texture, and storage testing. Accordingly, terms such as “network formation,” “more elastic matrix,” “improved texture,” and “sugar‐free” have been removed unless directly supported. The appropriate product description is “sucrose‐replaced” or “without added sucrose”; total sugars were not quantified.

A factorial or mixture design, sweetness‐equivalence and total‐sugar measurements, freezing‐point and frozen‐water‐fraction determinations, imaging of ice crystals and air cells, and paired instrumental and sensory/oral‐processing measurements are required to isolate ingredient effects. Such evidence would determine whether the higher force represents desirable body or excessive hardness and whether the lower drainage persists during storage.

### 3.5 Integrated Composition–Structure Interpretation

The composition results help place the texture findings in context, provided that they are not treated as causal or nutritional evidence. Total dietary fiber was modestly higher in the sucrose‐replaced powder (4.76% ± 0.21%) than in the control (4.27% ± 0.08%; *p* = 0.042). Reported protein was 0.14% ± 0.10% and 0.03% ± 0.00%, respectively (*p* = 0.197), while carbohydrate by difference was 97.62% ± 1.00% and 98.16% ± 1.00% (*p* = 0.545; Table 2). Thus, the broad protein and carbohydrate estimates did not distinguish the formulations statistically; the clearest measured composition difference was the approximately 0.49‐percentage‐point increase in total dietary fiber.

Across states, the sucrose‐replaced formulation combined modestly higher fiber and moisture with higher plotted viscoelastic moduli, lower plotted tan δ, greater back‐extrusion force, and lower drainage in the archived melting profile. Overrun remained close to 50%, so the higher instrumental resistance was not accompanied by a large change in incorporated air. This convergence is consistent with the broader frozen‐dessert literature, in which the serum phase, hydrocolloids, air, ice, fat or oil, and dispersed solids act together to govern rheology, deformation, and meltdown (VanWees et al. 2020; Sözeri Atik and Hartel 2025). In strawberry sorbet, hydrocolloid addition has also been shown to alter freezing behavior and ice‐crystal structure, reinforcing the need to treat formulation solids as part of the frozen matrix rather than as sweetness carriers alone (Kamińska‐Dwórznicka et al. 2020).

The pattern is therefore described as formulation‐level co‐variation rather than as a correlation between fiber and texture. Only two complete formulations were compared, several ingredient proportions changed together, and the rheology and melting data were available only as archived curves. The present evidence cannot determine whether the modest fiber difference, the replacement of low‐molecular‐weight sucrose, the much larger maltodextrin fraction, inulin, psyllium, oat solids, strawberry solids, or their interactions produced the higher force and lower drainage. Previous studies have likewise shown that protein source, stabilizer level, emulsifier type, and microstructural characteristics can affect rheological, textural, and melting responses in formulation‐dependent ways (VanWees et al. 2020; Sözeri Atik and Hartel 2025).

Protein is not a convincing explanation for the texture response. The reported values were small, the difference was not statistically significant, and the nitrogen‐to‐protein conversion factor was unavailable. By contrast, studies designed specifically as protein‐enrichment experiments added substantial quantities of whey or plant protein and paired composition changes with sensory or texture‐specific testing. Protein‐enriched frozen‐dessert formulations can exhibit formulation‐dependent sensory responses, including bitter or off‐flavor, gritty/grainy, gummy, cohesive, or adhesive perceptions, although protein addition does not necessarily reduce consumer acceptance (Ettinger et al. 2024; McDowell et al. 2022; Moss et al. 2023). The present sorbet should therefore not be described as protein‐enriched, high‐protein, or structured by a protein network.

The near‐equality of carbohydrate‐by‐difference estimates also does not mean that the carbohydrate systems were functionally equivalent. Sucrose, maltodextrin, inulin, psyllium‐associated fiber, and fruit‐ or oat‐derived carbohydrates differ in molecular size, solubility, freezing‐point depression, and interaction with water. These qualitative differences can influence the unfrozen serum phase even when a broad gravimetric remainder is similar. Fructan‐containing frozen desserts likewise demonstrate that replacing sugar or fat can change hardness, crystallized texture, viscosity, and melt perception, but the outcome depends on the whole formulation (Pintor‐Jardines et al. 2024). Because lipid and total sugars were not measured here, the carbohydrate remainder cannot establish reduced available carbohydrate or reduced total sugar.

For nutrition‐oriented product development, the most that can be said from these data is that a reconstitutable strawberry–oat premix can be formulated with sucrose replacement and plant‐derived fiber ingredients while maintaining rapid wetting and air incorporation. The results do not show that the prepared sorbet is healthier, lower in energy, lower glycemic, prebiotic, or eligible for a fiber claim. Such positioning requires serving‐based composition after reconstitution, total and added sugars, available carbohydrate, energy value, validated dietary‐fiber methods and regulatory thresholds, together with digestibility, glycemic, sensory, and intake evidence as appropriate.

## 4 Conclusions

Replacing sucrose with maltodextrin, inulin, and a commercial high‐intensity sweetener blend changed the strawberry–oat premix before and after reconstitution. The replacement powder still wetted rapidly, but it had a higher angle of repose and a lower compaction force. After dynamic freezing, the prepared sorbet showed a higher back‐extrusion force, a similar overrun, and lower drainage in the archived profile at 24°C. The modestly higher fiber value and the texture responses moved in a technologically coherent direction, but the two‐formulation design does not establish a statistical correlation or identify the contribution of any single ingredient. Protein and carbohydrate estimates did not support enrichment, reduced‐carbohydrate, or health claims. Taken together, the results reveal a powder–composition–texture trade‐off that matters when frozen desserts are designed as dry premixes. Microstructural imaging, confirmatory rheology, complete serving‐based nutritional composition, and sensory testing are needed before scale‐up or consumer‐facing claims are considered.

## Author Contributions

**Caroline dos Santos Lima:** conceptualization, investigation, methodology, data curation, writing – review and editing, writing – original draft, formal analysis, visualization. **Suzana Caetano da Silva Lannes:** supervision, writing – review and editing, project administration, resources, validation.

## Funding

This study was financed in part by the Coordenação de Aperfeiçoamento de Pessoal de Nível Superior—Brasil (CAPES)—Finance Code 001.

## Ethics Statement

The authors have nothing to report.

## Consent

The authors have nothing to report.

## Conflicts of Interest

The authors declare no conflicts of interest.

## Data Availability

Available data supporting the findings of this study may be obtained from the corresponding author upon reasonable request. The original rheometer files and replicate‐level melting time series are no longer available, as reported in the Methods.
